# Estimating illicit cigarette consumption using a tax-gap approach, India

**DOI:** 10.2471/BLT.20.251447

**Published:** 2020-08-27

**Authors:** Mark Goodchild, Thamizh Valavan, Praveen Sinha, Fikru Tesfaye Tullu

**Affiliations:** a Fiscal Policies for Health, Health Promotion Department, World Health Organization, 20 Avenue Appia, 1211 Geneva 27, Switzerland.; b Ministry of Finance, Government of India, New Delhi, India.; c World Health Organization Country Office in India, New Delhi, India.

## Abstract

**Objective:**

To estimate the magnitude of illicit cigarette consumption in India using a tax-gap approach.

**Methods:**

In the tax-gap analysis, illicit cigarette consumption in India was defined as the difference between total and legal consumption. Data on total cigarette consumption came from two national Global Adult Tobacco Surveys carried out from 2009 to 2010 and from 2016 to 2017. Legal consumption was derived from Government of India data on domestic cigarette production and trade.

**Findings:**

Estimated total cigarette consumption was 104.8 billion sticks in 2009 to 2010 and 94.2 billion sticks in 2016 to 2017, a decrease of 10.6 billion sticks, or of 10%, over the time period. Legal cigarette consumption fell from 99.4 to 88.5 billion sticks over the same period, a drop of 11%. Estimated illicit cigarette consumption was, therefore, 5.4 billion sticks in 2009 to 2010 and 5.6 billion sticks in 2016 to 2017, and accounted for 5.1% and 6.0% of the market in these periods, respectively. Consequently, only about 1 in 20 cigarettes consumed in India was illicit. Between 2016 and 2017, the estimated equivalent retail sales value of illicit cigarettes was 49 billion Indian rupees (753 million United States dollars, US$) and the estimated tax revenue foregone was 25 billion Indian rupees (US$ 390 million).

**Conclusion:**

Illicit cigarette consumption is relatively modest in India by global standards. Nonetheless, India should strengthen its capacity to control the illicit tobacco market as part of a comprehensive tobacco control strategy, while also continuing to implement traditional demand reduction measures, such as tobacco taxation.

## Résumé

**Objectif:**

Estimer l'ampleur de la consommation illicite de cigarettes en Inde grâce à une approche basée sur l'écart fiscal.

**Méthodes:**

Pour procéder à l'analyse de l'écart fiscal, la consommation illicite de cigarettes en Inde a été définie en calculant la différence entre la consommation totale et la consommation légale. Les données relatives à la consommation totale de cigarettes proviennent des résultats nationaux de deux Enquêtes mondiales sur le tabagisme chez l'adulte (GATS), menées de 2009 à 2010 et de 2016 à 2017. Celles sur la consommation légale ont été extraites des données du gouvernement indien concernant la production et le commerce de cigarettes dans le pays.

**Résultats:**

La consommation totale s'élevait à 104,8 milliards de cigarettes de 2009 à 2010, et à 94,2 milliards de cigarettes de 2016 à 2017. Ce qui représente une baisse de 10,6 milliards de cigarettes, donc de 10%, au cours de cette période. La consommation légale a chuté, passant de 99,4 à 88,5 milliards de cigarettes sur la même période, une diminution de 11%. Par conséquent, la consommation illicite est estimée à 5,4 milliards de cigarettes de 2009 à 2010, et à 5,6 milliards de cigarettes de 2016 à 2017. L'équivalent de 5,1% et 6,0% du marché, respectivement. Ainsi, sur l'ensemble des cigarettes consommées en Inde, seule une cigarette sur vingt environ était illicite. Entre 2016 et 2017, on considère que le commerce illicite de cigarettes a rapporté l'équivalent de 49 milliards de roupies indiennes (753 millions de dollars américains) de ventes au détail, tandis que le manque à gagner fiscal s'est élevé à 25 milliards de roupies indiennes (390 millions de dollars américains).

**Conclusion:**

La consommation illicite de cigarettes est relativement modeste en Inde par rapport aux standards internationaux. Néanmoins, le pays devrait renforcer ses capacités de contrôle du commerce illicite du tabac dans le cadre d'une stratégie complète de lutte contre le tabagisme, tout en continuant à mettre en œuvre les mesures traditionnelles visant à réduire la demande, comme la taxation du tabac.

## Resumen

**Objetivo:**

Estimar la magnitud del consumo ilícito de cigarrillos en la India mediante la aplicación de un enfoque de diferencias impositivas.

**Métodos:**

El consumo ilícito de cigarrillos en la India se definió en el análisis de la diferencia impositiva como la diferencia entre el consumo total y el consumo legal. Los datos sobre el consumo total de cigarrillos procedían de dos Encuestas mundiales sobre el tabaquismo en adultos de alcance nacional que se realizaron entre 2009 y 2010 y entre 2016 y 2017. El consumo legal se obtuvo a partir de los datos del Gobierno de la India sobre la producción y el comercio nacional de cigarrillos.

**Resultados:**

Se estima que el consumo total de cigarrillos fue de 104 800 millones de cigarrillos entre 2009 y 2010 y 94 200 millones de cigarrillos entre 2016 y 2017, lo que supone una reducción de 10 600 millones de cigarrillos, o del 10 %, durante ese periodo. El consumo legal de cigarrillos se redujo de 99 400 a 88 500 millones de cigarrillos en el mismo periodo, un descenso del 11 %. Por lo tanto, el consumo ilícito de cigarrillos estimado fue de 5400 millones de cigarrillos entre 2009 y 2010 y de 5600 millones de cigarrillos entre 2016 y 2017, lo que representó el 5,1 % y el 6,0 % del mercado en estos periodos, respectivamente. Por consiguiente, solo 1 de cada 20 cigarrillos que se consumen en la India es ilícito. Entre 2016 y 2017, el valor equivalente estimado de las ventas al por menor de cigarrillos ilícitos fue de 49 000 millones de rupias indias (753 millones de dólares estadounidenses, USD) y el estimado de los ingresos fiscales no percibidos fue de 25 000 millones de rupias indias (390 millones de USD).

**Conclusión:**

El consumo ilícito de cigarrillos es relativamente moderado en la India según las normas mundiales. No obstante, la India debería fortalecer su capacidad para controlar el mercado ilícito de tabaco como parte de una estrategia integral de control del tabaco, al tiempo que continúa aplicando medidas tradicionales para reducir la demanda, como los impuestos sobre el tabaco.

## ملخص

الغرض تقدير حجم الاستهلاك غير المشروع للسجائر في الهند باستخدام نهج الفجوة الضريبية.

الطريقة في تحليل للفجوة الضريبية، تم تعريف الاستهلاك غير المشروع للسجائر في الهند على أنه الفرق بين الاستهلاك الإجمالي والاستهلاك القانوني. جاءت البيانات المتعلقة بالاستهلاك الإجمالي للسجائر من مسحين وطنيين عالميين عن التبغ للبالغين، تم إجراؤهما في الفترة من 2009 إلى 2010، ومن 2016 إلى 2017. وتم اشتقاق الاستهلاك القانوني من بيانات الحكومة الهندية حول إنتاج وتجارة السجائر المحلية على الصعيد المحلي.

النتائج بلغ تقدير إجمالي استهلاك السجائر 104.8 مليار سيجارة في الفترة من 2009 إلى 2010، و94.2 مليار سيجارة في الفترة من 2016 إلى 2017، بانخفاض قدره 10.6 مليار سيجارة، أو ما يعادل نسبة 10٪، خلال الفترة الزمنية. انخفض الاستهلاك القانوني للسجائر من 99.4 إلى 88.5 مليار سيجارة خلال نفس الفترة، بانخفاض قدره 11٪. وبالتالي بلغ تقدير الاستهلاك غير المشروع للسجائر 5.4 مليار سيجارة في الفترة من 2009 إلى 2010، و5.6 مليار سيجارة في الفترة من 2016 إلى 2017، بانخفاض قدره 5.1%، و6.0%، من السوق خلال هاتين الفترتين على التوالي. وبالتالي ، فإن 1 من كل 20 سيجارة يتم تدخينها في الهند كانت غير مشروعة. بين عامي 2016 و2017، كانت القيمة التقديرية لمبيعات التجزئة المعادلة للسجائر غير المشروعة 49 مليار روبية هندية (753 مليون دولارًا أمريكيًا)، وبلغت الإيرادات الضريبية التقديرية السابقة 25 مليار روبية هندية (390 مليون دولارًا أمريكيًا).

الاستنتاج يعتبر الاستهلاك غير المشروع للسجائر متواضع نسبيًا في الهند بالمعايير العالمية. وبالرغم من ذلك، يجب على الهند تعزيز قدرتها على التحكم في سوق التبغ غير المشروع كجزء من استراتيجية شاملة لمكافحة التبغ، مع الاستمرار كذلك في تنفيذ تدابير تقليص الطلب التقليدية، مثل فرض الضرائب على التبغ.

## 摘要

**目的:**

使用税收缺口法估算印度的非法卷烟消费量规模。

**方法:**

在税收缺口法中，印度的非法卷烟消费量是指总消费量与合法消费量之间的差额。卷烟总消费量的数据来自于 2009-2010 年和 2016-2017 年在全国范围内进行的两次“全球成人烟草调查”。合法消费量来源于印度政府关于国内卷烟生产和贸易的数据。

**结果:**

据估计，2009-2010 年卷烟总消费量为 1048 亿支，2016-2017 年为 942 亿支，同比减少 106 亿支，降幅为 10%。同期合法卷烟消费量从 994 下降至 885 亿支，下降了 11％。因此，据估计，2009-2010 年非法卷烟消费量为 54 亿支，2016-2017 年为 56 亿支，分别占同期市场份额的 5.1% 和 6.0%。因此，在印度消费的卷烟中，只有大约二十分之一是非法的。2016 年至 2017 年，非法卷烟的等值零售价值估计为 490 亿印度卢比（7.53 亿美元），税收损失估计为 250 亿印度卢比（3.9 亿美元）。

**结论:**

按全球标准衡量，印度的非法卷烟消费量相对适中。尽管如此，作为全面烟草控制战略的一部分，印度应加强其控制非法烟草市场的能力，同时还应继续执行烟草税等传统的减少需求措施。

## Резюме

**Цель:**

Оценка масштабов незаконного потребления сигарет в Индии с использованием подхода налогового разрыва.

**Методы:**

В анализе налоговых разрывов незаконное потребление сигарет в Индии было определено как разница между общим и законным потреблением. Данные об общем потреблении сигарет были получены из двух национальных глобальных опросов потребления табачных изделий среди взрослого населения, проведенных с 2009 по 2010 г. и с 2016 по 2017 г. Данные о законном потреблении были получены из данных правительства Индии по внутреннему производству сигарет и торговле ими.

**Результаты:**

Предполагаемое общее потребление сигарет составило 104,8 млрд штук за период 2009–2010 гг. и 94,2 млрд штук за период 2016–2017 гг., что на 10,6 млрд штук, или на 10%, меньше за указанный период. За тот же период законное потребление сигарет упало с 99,4 до 88,5 млрд штук, т. е. на 11%. Таким образом, предполагаемое незаконное потребление сигарет составило 5,4 млрд штук за период 2009–2010 гг. и 5,6 млрд штук за период 2016–2017 гг.; на эти периоды приходились доли 5,1% и 6,0% рынка соответственно. Следовательно, только одна из двадцати сигарет, которые потреблялись в Индии, была незаконной. В период с 2016 по 2017 г. оценочный эквивалентный стоимостный объем розничных продаж незаконных сигарет составил 49 млрд индийских рупий (753 млн долларов США), а предполагаемые упущенные налоговые поступления в бюджет составили 25 млрд индийских рупий (390 млн долларов США).

**Вывод:**

Уровень незаконного потребления сигарет в Индии относительно низкий по мировым стандартам. Тем не менее Индии следует усилить свой потенциал по контролю над незаконным табачным рынком в рамках всеобъемлющей стратегии борьбы против табака, а также продолжить реализацию традиционных мер по сокращению спроса, таких как налогообложение табачных изделий.

## Introduction

The widespread availability of illicit tobacco products can undermine the effectiveness of both public health interventions, such as health warnings on tobacco packs, and fiscal measures, such as tobacco taxation. Measures to counter illicit trade in these products should be part of any comprehensive tobacco control strategy and many countries devote considerable resources to tackling such illicit markets. Moreover, global efforts to address illicit tobacco, in particular, have led to the adoption of the Protocol to Eliminate Illicit Trade in Tobacco Products, which was produced under the World Health Organization’s (WHO’s) Framework Convention on Tobacco Control.^1^ The protocol came into force on 25 September 2018 and commits signatory parties to implement a range of measures on illicit trade designed to make tobacco control interventions more effective, while also serving to protect government revenue from the corrosive effects of that trade.

Many countries recognize the need to produce more reliable estimates of the magnitude of the illicit tobacco market. Such estimates can help in monitoring both the impact of measures specified by the protocol to control the illicit market and the overall effectiveness of all tobacco control efforts in curbing consumption. Globally, it has been estimated that illicit cigarettes account for 11.6% of the total cigarette consumption.^2^ However, empirically sound estimates of the size of the illicit market in developing countries, such as India, are still relatively scarce. Only one independent, peer-reviewed study of the illicit cigarette market in India has been carried out:^3^ in 2018, John and Ross used an empty-pack survey approach, which has often been used in academic studies elsewhere, and found that 2.7% of 11 063 empty packs collected from 1727 retailers across India were illicit. They found that illicit cigarette consumption in India was generally low, though there was some regional variation, with Aizawl near the Bangladesh and Myanmar borders recording a relatively high proportion of illicit cigarettes. The study’s findings contrasted sharply with the tobacco industry’s claim that the proportion of illicit cigarettes on India’s market increased from 15 to 24% between 2010 and 2015.^4^

Reliable estimates of the magnitude of the illicit tobacco market can be obtained by either: (i) survey-based approaches, such as studying empty packs; (ii) econometric modelling of demand; or (iii) undertaking a so-called tax-gap analysis of the difference between the total consumption (often based on a household survey) and the consumption derived from the level of sales on which tax was paid.^5^^,^^6^ The aim of our study was to use the tax-gap approach to estimate the magnitude of illicit cigarette consumption in India using data from the country’s Global Adult Tobacco Survey in 2016 to 2017 (GATS II).^7^ In addition, we repeated the analysis with data from a similar survey carried out in 2009 to 2010 (GATS I),^8^ thereby generating results for two time points using the same method.

## Methods

A tax-gap analysis designed to establish the total consumption of both legal and illicit cigarettes generally relies on the existence of a household survey, as well as on government tax data. In India, Global Adult Tobacco Surveys (GATSs) are nationally representative household surveys of adults that are systematically carried out in all 30 states and in the two union territories of Chandigarh and Puducherry: (i) GATS I was undertaken in 2009 to 2010, involved 69 296 completed interviews and had an overall response rate of 91.8%;^8^ and (ii) GATS II was carried out in 2016 to 2017, involved 74 037 interviews and had an overall response rate of 92.9%.^7^

In the tax-gap analysis we used, annual illicit cigarette consumption, *C_i_*, which was calculated as the annual total cigarette consumption, *C_t_*, derived from the GATS minus the annual legal consumption, *C_l_*, derived from Government of India data on domestic cigarette production and trade: 
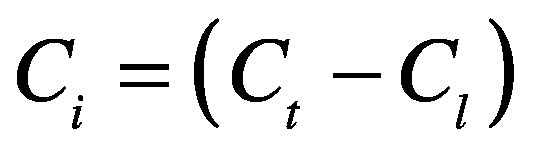
. To avoid inaccuracies potentially caused by the inventory and front-loading practices of the tobacco industry, we used the average *C_l_* for the 3 years around each GATS; for example, the estimated legal consumption corresponding to GATS II in 2016 and 2017 was the average *C_l_* for 2016, 2017 and 2018.

 We also used earlier cigarette consumption and duty revenue data dating as far back as 1996-97 to highlight several long-term trends of general interest.^9^ Specifically, legal cigarette consumption was converted into a per capita basis to adjust for India’s population growth, while annual duty revenue was expressed in constant 2017 price terms to adjust for inflation. India’s population and inflation statistics was sourced from the IMF’s World Economic Outlook.^10^

Annual cigarette consumption was calculated by multiplying the total number of current male and female cigarette smokers, *S_t_*, by the average daily consumption, *D_d_*, of cigarette smokers for each gender, *g*, as derived from GATS I and GATS II data:
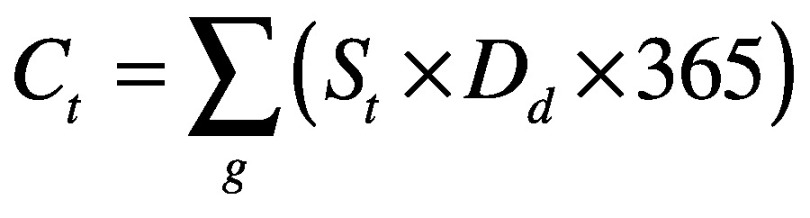
(1)To address possible uncertainty in the survey data, we carried out a sensitivity analysis by varying total annual cigarette consumption by 5% around the median estimate.

The Government of India periodically publishes data on the seizure of illegal tobacco products, including their estimated retail sales value. The value of seizures averaged 1696 million Indian rupees (₹; 26 million United States dollars, US$) over the GATS II survey period: ₹1504 million (US$ 23 million) in 2015 to 2016, ₹1933 million (US$ 30 million) in 2016 to 2017 and ₹1650 million (US$ 25 million) in 2017 to 2018. These estimates also provide an opportunity to explore the success of India’s counter-smuggling and other illicit tobacco control activities.^11^

Quantifying the equivalent retail value of all illicit cigarettes in aggregate can be difficult, because the selling price can vary. Indeed, evidence from other countries shows that illicit cigarettes are often more, rather than less, expensive than legal offerings.^12^ In broad terms, however, the equivalent retail sales value of illicit cigarettes can be estimated by multiplying the number of illicit cigarette sticks by their average retail price in the legal, retail market. In India, the average retail price of 10 cigarette sticks was reported to be about ₹87 (US$ 1.34) in 2016 to 2017.^13^^,^^14^

Lastly, we can estimate the amount of tax revenue forgone due to illicit cigarette consumption from the average tax paid on a cigarette pack in the legal market. In 2016 to 2017, central government duty accounted for an average of ₹25(US$ 0.38) on each pack of 10 cigarettes, whereas state value added tax accounted for ₹20 (US$ 0.31).^13^^,^^14^ Hence, the average tax was about ₹45 (US$ 0.69) on each pack of 10 cigarettes in 2016 to 2017, or around 52% of the retail price.

## Results

Table 1 shows data on annual cigarette consumption in India during 2009 to 2010 and 2016 to 2017, partially derived from GATS I and GATS II, respectively. Between these time periods, the prevalence of cigarette smoking in the country fell significantly by around 30%, from 5.7% to 4.0% of the adult population (*P* < 0.01).^7^ Correspondingly, the number of cigarette smokers fell by around 19%, from 46.4 million to 37.5 million; there were 8.8 million fewer cigarette smokers in 2016 to 2017 compared with 2009 to 2010. On the other hand, smoking intensity increased by around 10%, from 6.2 to 6.8 sticks per smoker daily. Thus, the estimated total (i.e. legal and illicit) cigarette consumption decreased by around 10% from 104.8 billion sticks in 2009 to 2010 to 94.2 billion in 2016 to 2017, which represents 10.6 billion fewer cigarettes consumed by India’s smokers annually.

**Table 1:** Legal and illicit cigarette consumption, India, 2009–2010 and 2016–2017

Cigarette consumption measure	2009–10^a^	2016–17^a^	Absolute (%) change between time periods
Proportion of adult population who smoked, %	5.7	4.0	−1.7 (−30)
No. adult smokers, millions	46.4	37.5	−8.8 (−19)
Average sticks smoked per day, no. per smoker	6.2	6.8	+0.60 (+10)
Total sticks consumed, billions	104.8	94.2	−10.6 (−10)
Legal sticks consumed, billions	99.4	88.5	−10.9 (−11)
Illicit sticks consumed, billions	5.4	5.6	+0.3 (+5)
Illicit market share, %	5.1	6.0	+0.9 (+17)
Total sticks consumed per capita annually, no.	128.9	100.3	−28.5 (−22)
Legal sticks consumed per capita annually, no.	122.2	94.3	−27.9 (−23)
Illicit sticks consumed per capita annually, no.	6.6	6.0	−0.6 (−9)

^a^ Figures were derived from Global Adult Tobacco Surveys I and II, which were carried out in India during 2009 to 2010 and 2016 to 2017, respectively, and from the Government of India data on domestic cigarette production and trade.

**Fig. 1:**
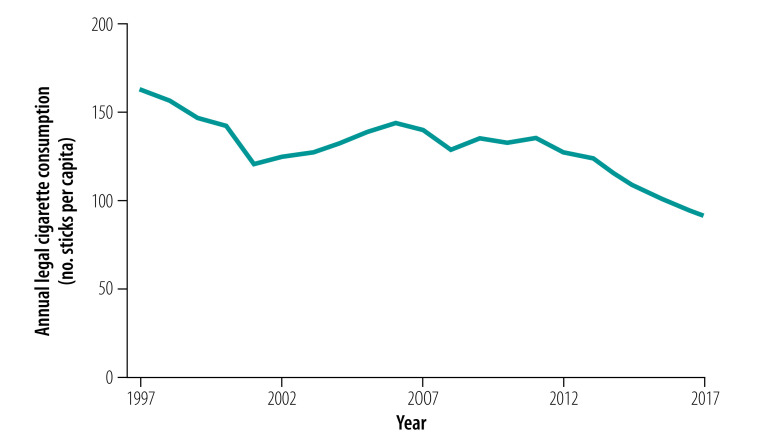
Annual legal cigarette consumption per capita, India, 1997–2017

Note: Data were obtained from the Government of India.^9^

**Fig. 2:**
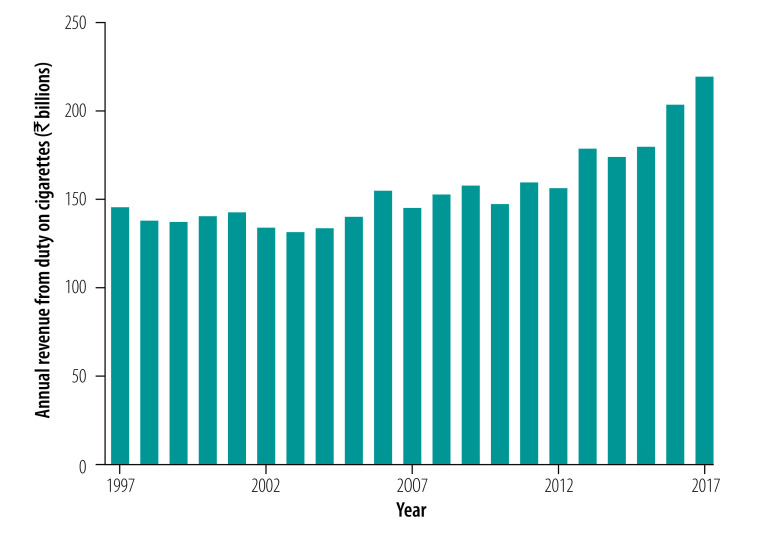
Annual government revenue from duty on cigarettes, India, 1997–2017

Notes: Data were obtained from the Indian Ministry of Finance.^10^ The graph shows revenue in constant 2017 Indian rupees (₹).1 USD = 65.12 INR.

Similarly, estimated legal cigarette consumption decreased by around 11% from 99.4 billion sticks in 2009 to 2010 to 88.5 billion sticks in 2016 to 2017, which represents 10.9 billion fewer legal cigarettes consumed annually. This decrease is consistent with the long term trend for legal cigarette consumption on a per capita basis, which has fallen by 44% from 163 to 91 sticks per year between 1997 and 2017 (Fig 1).Over the same two decade period, annual revenue from duty on cigarettes has increased by 50% from 146 to almost 220 billion Indian rupees (US$ 2,244 to 3,376 million) after adjusting for inflation (Fig 2).

Using the figures for total and legal cigarette consumption in 2016 to 2017, we estimated that illicit cigarette consumption was 5.6 billion sticks in that period, which was 6.0% of the total cigarette consumption. By comparison, estimated illicit cigarette consumption in the earlier GATS I period from 2009 to 2010 was 5.4 billion sticks, or 5.1% of the total cigarette consumption. Overall, our findings suggest that only about 1 in 20 cigarettes in India was illicit in both time periods. Using an average retail price of legal cigarettes of ₹87 per pack of 10 in 2016 to 2017, we estimated that the retail value of illicit cigarettes in that period was ₹49 billion (US$ 753 million), including foregone tax revenues of around ₹25 billion (US$ 390 million). Government data on the seizure of illegal tobacco products suggest that less than 5% of illicit cigarettes were captured by existing countermeasures against illicit trade.^11^

Table 2 shows the findings of our sensitivity analysis of the total cigarette consumption: we varied the median estimated consumption from GATS I and GATS II by plus and minus 5%. This resulted, for example, in a range for the total illicit consumption in 2016 to 2017 from 0.91 to 10.33 billion sticks around the 5.6 billion sticks median. Consequently, the estimated market share of illicit cigarettes in India ranged from 1.0% to 10.5% around a median of 6.0%. From a public finance perspective, these estimates suggest that the tax revenue foregone in India in 2016 to 2017 ranged between ₹4 billion and 47 billion (US$ 63 to 715 million). Although estimated illicit cigarette consumption in India increased from 5.1% to 6.0% of the total cigarette consumption between 2009 to 2010 and 2016 to 2017, our sensitivity analysis shows that the directional pattern is not certain and there could, in fact, have been a decrease. A decrease would be consistent with the significant 30% drop in smoking prevalence in the country (*P* < 0.01).^7^

**Table 2:** Sensitivity analysis of illicit cigarette consumption, India, 2009–2010 and 2016–2017

Illicit cigarette consumption measure^a^	Sensitivity analysis^b^	2009–10^b^	2016–17^b^	Absolute change between time periods	
Illicit sticks consumed, billions	Upper estimate	10.6	10.3	−0.29	
	Median estimate	5.4	5.6	+0.26
	Lower estimate	0.1	0.9	+0.77
Illicit market share, %	Upper estimate	9.7	10.5	+0.79	
	Median estimate	5.1	6.0	+0.85
	Lower estimate	0.1	1.0	+0.88

^a^ Illicit cigarette consumption was the difference between total cigarette consumption (from Global Adult Tobacco Surveys I and II carried out during 2009 to 2010 and 2016 to 2017, respectively) and legal consumption (from the Government of India data on domestic cigarette production and trade).

^b^ In the sensitivity analysis, the estimated total annual cigarette consumption, from which illicit consumption was derived, was varied by plus and minus 5% around the median.

## Discussion

Our analysis found that the estimated illicit cigarette market in India accounted for 6.0% of the total cigarette consumption in 2016 to 2017. This result is consistent with that of John and Ross, who found that illicit cigarettes accounted for 2.7% of the total cigarette market in India in 2016.^3^ Our findings also indicate that the illicit market in India is relatively modest compared to the global estimated average of 11.6% in 2009,^2^ and certainly well below the figure of 24% estimated by the tobacco industry in 2015.^4^ Nonetheless, it is important that the government addresses illicit tobacco consumption for both health and fiscal reasons. As illicit products are unregulated, they lack health warnings and the absence of taxation makes them cheaper and more accessible for vulnerable groups, such as young and poor people. Tobacco use accounts for 11% of all deaths in India, there are close to one million tobacco-related deaths each year.^15^ Consequently, countermeasures against illicit consumption should be an integral component of any comprehensive tobacco control strategy. India is a signatory to both WHO’s Framework Convention on Tobacco Control and the Protocol to Eliminate Illicit Trade in Tobacco Products and has, therefore, committed to increasing efforts to tackle the illicit tobacco market.^1^^,^^16^ Moreover, there may be a large fiscal advantage to enhancing India’s capacity to tackle this market. Experience from across the world and in developing countries, such as Brazil, Kenya and Turkey, has shown that countermeasures, such as tracking and tracing systems, vendor licensing and higher penalties, can protect and even increase revenue collection.^5^

Our analysis also suggests that per capita, cigarette consumption in India decreased by 22% between GATS I and GATS II. Similarly, the number of legal cigarette sticks consumed per capita fell by 23% over this period. This result is consistent with the longer-term trend decrease in per capita consumption.^9^ Tobacco control activities, such as bans on advertising and promotion, public awareness campaigns and smoke-free areas might have reduced the demand for tobacco products. Nevertheless, government revenue from the duty on cigarettes has continued to rise particularly in recent years, despite the decrease in consumption.^10^ Indeed, annual revenue from cigarette duty increased by almost 50% in constant-price terms between 2009 to 2010 and 2016 to 2017.^17^^,^^18^ This increase in revenue was due to higher duty rates, particularly in the past decade when the average duty almost doubled in inflation-adjusted terms. This win–win effect is a common theme in many developing countries, including large countries like Brazil, the Philippines, South Africa, Turkey and Ukraine, where higher tobacco taxes have contributed to both fiscal and public health targets.^5^

One unique feature in India is the extensive use of so-called indigenous tobacco products, such as the *bidi*, which is the dominant form of smoking.^7^ A concern sometimes raised in the context of India is that the large decrease seen in cigarette use may have been due to smokers shifting to *bidis*. However, the evidence shows that cigarettes and *bidis* appeal to very different consumer markets. The cross-price elasticity of demand is very weak, at −0.05 (95% confidence interval: −0.10 to 0.01), which means that little switching between products will occur in response to a price change alone. Indeed, the negative sign on the coefficient suggests, if anything, that cigarettes and *bidis* are complementary products rather than substitutes.^19^^,^^20^ In addition, GATS II showed that the use of all forms of smoked tobacco in India, including *bidis*, had decreased significantly since 2009 to 2010 (*P* < 0.01). Nevertheless, *bidi* use decreased less steeply: the relative decline was 16% compared to 30% for cigarettes.^7^ One factor that might explain this difference is affordability. For example, a recent study found that *bidis* have remained very affordable over the past decade, whereas cigarettes have become less affordable.^21^

The tax treatment of *bidis* remains very lenient in India, even under the new Goods and Services Tax system introduced in 2017.^22^ In fact, tax accounted for just 22% of the retail price of *bidis* in 2018 compared to more than 50% for cigarettes and smokeless tobacco products.^13^ Hence, a substantial rise in taxes on *bidis* is needed to promote smoking cessation more widely across the population. In contrast, India has made steady progress with the tax treatment of cigarettes, as shown in Fig. 2. However, the government has maintained a tiered tax system for cigarettes, whereby the tax rate differs according to the length of the cigarette and the filter status of each brand. Such tiered systems can be manipulated by manufacturers and are, therefore, associated with lower tax yields and cigarette prices.^5^^,^^23^ Evidently, India’s tiered system has allowed manufacturers to promote the shortest and cheapest brands, which has led to an expansion in the market share of short (i.e. less than 65 mm) cigarettes.

Such actions by the industry undermine the overall effectiveness of tobacco taxation by encouraging the uptake of cheaper cigarettes, particularly by price-sensitive consumers, such as teenagers and young adults. For this reason, WHO recommends countries adopt a uniform system of taxation, whereby every brand is subject to the same tax rates or follows the same rules.^5^^,^^23^ India should move towards a uniform tax system for cigarettes as quickly as possible.

The tax-gap approach we used here has been used widely throughout the world to estimate the size of illicit cigarette markets,^5^ partly because it is relatively easy to piggy-back off existing surveys, such as a GATS. Moreover, GATSs are designed to be nationally representative, which is an important consideration in heavily populated countries like India, where a separate nationally representative survey on the illicit tobacco trade could be an expensive undertaking. Some 30 countries have already conducted GATSs and a number, such as India, have completed several rounds. In many other countries, data on smoking behaviour can be obtained from national censuses or targeted surveys of health or household expenditure. In the future, it may be feasible to insert questions about illicit consumption directly into a GATS or similar questionnaire.

Another strength of the tax-gap approach is that findings can be framed against the background of aggregate changes in smoking behaviour, particularly smoking prevalence. This feature is perhaps most relevant when data from multiple surveys are available or when aggregate demand changes substantially. It is important to recognize that other methods for estimating illicit trade lack this perspective and can therefore be misleading, particularly when seizure statistics are used. We estimated, for example, that seizures accounted for less than 5% of illicit consumption in India. Hence, even a large change in the quantity of seizures will not necessarily give an accurate picture of the underlying trend in illicit consumption.

One limitation of the tax-gap approach is the degree of uncertainty in even nationally representative surveys. We addressed this issue, in part, by conducting a sensitivity analysis. A more important limitation is that several studies in high-income countries indicate that survey respondents may under-report cigarette use because of the social stigma attached to smoking.^24^^,^^25^ Other studies suggest that infrequent smokers understate their smoking intensity more than frequent smokers.^26^ We have not made any adjustment in our study to reflect these possibilities, as such adjustments tend to be arbitrary in any case. We simply let the GATS results speak for themselves. In addition, as 29% of adults and 42% of men still use tobacco products in India, it is difficult to imagine there is a high level of social stigmatization linked to tobacco use in the country, at least not the level experienced in many high-income countries.

Another limitation is that, although the tax-gap approach can give an estimate of the overall size of the illicit market, it provides little detail. Such detail is helpful for several reasons. First, understanding the types and sources of illicit tobacco products is important so that, for example, resources can be devoted to countering the market more effectively. In India, it has traditionally been thought that the illicit market is mainly supplied by small domestic producers (i.e. illegal manufacturing) rather than by cross-border smuggling. A source of information other than a tax-gap analysis may be needed to track changes in the sources of illicit trade. Second, it is important to have a good understanding of the socioeconomic characteristics of illicit consumers, of the prices at which they purchase illicit products and of any brand preferences. Such information can be gathered using other methods, most notably surveys of smokers themselves or of empty or discarded packs. Consequently, researchers could gain a better understanding of the demand for illicit cigarettes by developing a hybrid approach that combines a tax-gap analysis with other methods.^5^ For example, recent studies in Brazil, the Gambia and Malaysia have used GATS data in combination with information about minimum legal prices to assess the share of cheap illicit cigarettes in the market.^27^^–^^29^ Such methods can improve traditional tax-gap analyses by tailoring them to the individual country’s conditions.

In conclusion, our study found that illicit cigarette consumption was relatively modest in India, certainly by global standards. Nonetheless, addressing illicit tobacco as a matter of good risk management is important from both health and fiscal perspectives. India should strengthen its capacity to control illicit trade in tobacco as part of a comprehensive tobacco control strategy, while also continuing to implement traditional demand reduction measures, such as tobacco taxation.
